# The pattern of coding sequences in the chloroplast genome of *Atropa belladonna* and a comparative analysis with other related genomes in the nightshade family

**DOI:** 10.5808/gi.22045

**Published:** 2022-12-26

**Authors:** Satyabrata Sahoo, Ria Rakshit

**Affiliations:** 1 Department of Physics, Dhruba Chand Halder College, Dakshin Barasat 743372, India; 2 Department of Botany, Baruipur College, Baruipur 743610, India

**Keywords:** *Atropa belladonna*, codon bias, codon usage, chloroplast genome, medicinal plants

## Abstract

*Atropa belladonna* is a valuable medicinal plant and a commercial source of tropane alkaloids, which are frequently utilized in therapeutic practice. In this study, bioinformatic methodologies were used to examine the pattern of coding sequences and the factors that might influence codon usage bias in the chloroplast genome of *Atropa belladonna* and other nightshade genomes. The chloroplast engineering being a promising field in modern biotechnology, the characterization of chloroplast genome is very important. The results revealed that the chloroplast genomes of *Nicotiana tabacum, Solanum lycopersicum, Capsicum frutescens, Datura stramonium, Lyciumbarbarum, Solanum melongena*, and *Solanum tuberosum* exhibited comparable codon usage patterns. In these chloroplast genomes, we observed a weak codon usage bias. According to the correspondence analysis, the genesis of the codon use bias in these chloroplast genes might be explained by natural selection, directed mutational pressure, and other factors. GC12 and GC3S were shown to have no meaningful relationship. Further research revealed that natural selection primarily shaped the codon usage in *A. belladonn*a and other nightshade genomes for translational efficiency. The sequencing properties of these chloroplast genomes were also investigated by investing the occurrences of palindromes and inverted repeats, which would be useful for future research on medicinal plants.

## Introduction

*Atropa belladonna* is a common ingredient in homeopathy and other complementary and alternative medicine. It is a member of the nightshade family (*Solanaceae*). The plant's leaves and roots are mostly used to create medications, and it is said to be a major source of tropane alkaloids, as well as scopolamine and hyoscyamine [1]. Tropane alkaloids, which are found mostly in the *Solanaceae* family, are antimuscarinic medications that act primarily on the parasympathetic nervous system and are utilized as anticholinergic treatments in clinical practice. *A. belladonna* has a strong morphogenetic potential. It has been studied in vitro as a model system for the production of tropane alkaloids as well as the development of other alkaloids in diverse cultures [2]. Despite its reputation for being poisonous, research has revealed that the plant can be used for a variety of medical purposes. The availability of this plant's complete chloroplast genome sequencing could contribute to the development of current genetics and molecular biology by allowing researchers to better comprehend the expression of functional proteins. Plant chloroplasts are crucial organelles that mediate photosynthesis, intercellular signaling, and function as stress sensors from the outside environment [3,4]. On average, chloroplast genomes contain a small number of genes involved mostly in energy production and metabolic processes. High level of transgene expression is possible with chloroplast and thus, a very high level of gene expression and large scale of protein production is possible with chloroplast engineering. Exploring the molecular mechanism governing the expression of *A. belladonna* chloroplast genes can thus help to further the genetic approach to modern biotechnology. The purpose of this research is to study the compositional signature and to investigate how it affects codon use bias (CUB) in chloroplast genes.

CUB is the differential use of some preferred codons expressing the same amino acid in a protein-coding gene relative to others. The use of synonymous codons in the organization of genetic codes in a genomic DNA sequence has been proven to have a significant impact on the efficiency of mRNA translation and the accuracy of protein synthesis. It plays a crucial function in gene development and expression. There are various hypotheses on the mechanisms that determine the CUB of gene sequences [5]. Mutational pressure [6], natural selection [7], protein secondary structures, length of the protein-coding genes, aromaticity, and hydropathy of encoded proteins, and many other variables determine codon bias [8]. According to earlier studies [9,10], codon biases [11,12] are primarily created by an interplay between mutation pressure [13] and selection [14,15] constraints in many plant species. The codon bias in the Porphyra umbilicalis chloroplast genome [16] and the variability of codon usage patterns in the rice genome [17] are the consequence of a complex combination of natural selection, directed mutational bias, and nucleotide base compositions. Selection pressure, on the other hand, outnumbers mutation pressure in determining the codon use pattern in the cotton genome [18]. According to these studies, the factors that cause codon bias vary by plant species. The study of codon usage patterns may help to identify the primary driving forces. The codon bias of a gene and its expression is thought to be inextricably linked. As a result, looking at codon usage patterns could lead to new techniques for predicting and designing highly expressed genes [19-26]. The regulation of gene expression plays a central role in defining cell fates and controlling biological functions. The utilization of codons in highly expressed genes is frequently characterized by a substantial compositional bias. Several numerical indices have been established to objectively evaluate the degree of gene expression to predict highly expressed (PHE) genes [27,28]. The use of codon optimization could give crucial information about how to make synthetic genes that are highly expressed.

Here, we comprehensively examined synonymous codon usage patterns in the genes of chloroplast genomes in the nightshade family to determine the general patterns and influencing factors of codon bias in chloroplast genomes of *A. belladonna* and others (*Nicotiana tabacum, Capsicum frutescens, Solanum lycopersicum, Datura stramonium, Solanum melongena, Lycium barbarum*, and *Solanum tuberosum*). The purpose of this work is to investigate codon usage patterns using a variety of codon bias indicators. This research is important for understanding the molecular evolution and structural organization of chloroplast genes.

## Methods

The complete chloroplast genome of *A. belladonna* (NC_004561.1), together with all annotated genes, were obtained from the GenBank database of NCBI (http://www.ncbi.nlm .nih.gov/). The other chloroplast genome sequences of the nightshade family considered in the present study have Gene Bank accession numbers: NC_028007.1 (*C. frutescens*), NC_018117.1 (*D. stramonium*), NC_041110.1 (*L. barbarum*), NC_001879.2 (*N. tabacum*), NC_007898.3 (*S. lycopersicum*), KU682719.1 (*S. melongena*), and NC_008096.2 (*S. tuberosum*). Only the coding sequences with translatable codons, and with start and stop codons have been considered for the analysis.

### Analysis of base composition

We calculated the frequency of occurrence of nucleotides at three codon sites, the overall GC content, the GC content at the first (GC1), second (GC2), and third (GC3) codon position, and the frequency of occurrence of nucleotides of synonymous codons at the third position (A3_s_, T3_s_, G3_s_, and C3_s_) as a part of our analysis of codon usage pattern in the chloroplast genome. Mutations should happen at random at any codon site if there is no external pressure; otherwise, they will happen in a specified direction. If selection pressure exists, preference for a given base will differ at three codon sites; otherwise, the base composition will be similar at all three codon sites. In the absence of any external pressure from mutation or selection, the codon usage pattern follows the parity rule. The PR2 plot is employed to look for departures from the parity criteria. To visualize the distribution of four bases at the synonymous codon site, [A3/(A3 + T3)] assessing AT-bias is plotted against [G3/(G3 + C3)] measuring GC-bias [29] in the PR2 plot. The distribution of points around the center point reveals the degree of codon bias and its direction. Any significant deviation in the genome's base organization indicates that selection pressure is prevailing over mutation. The relative influence of mutation and selection on the formation of codon usage pattern can be determined by calculating GC3S (GC content at the synonymous site) and GC12, the average of GC1 and GC2 (GC content at the first codon site and second codon site, respectively). To investigate the impact of mutation pressure and selection pressure on codon usage, a regression line is created between GC12 and GC3S (neutrality plot). The slope of the line in the neutrality plot can reveal the relative influence of the mutational force on synonymous codon bias. If the slope of the line is zero, it indicates that directed mutation pressure has no effect. A slope of one, on the other hand, denotes perfect neutrality [24].

### Analysis of codon usage

The codon bias of a gene is generally measured by the relative synonymous usage of codons. It is measured as the usage of each codon compared to the average usage of synonymous codons in a set of protein-coding genes.

The Codon Adaptation Index (CAI), a measure of biasness of a gene based on the relative synonymous codons usage (RSCU) is given by Sharp and Li [27],


$$CAI={\left(\prod_{1}^{N}w_{i}\right)}^{\frac{1}{N}}$$

N is the length of a gene in terms of codon count and *w_i_* is relative adaptiveness of i^th^ codon in the gene. *w_i_* is defined as


$$w_{i}=\frac{{\left(RSCU\right)}_{i}}{{\left(RSCU\right)}_{i,max}}$$

where the following equation is used for the calculation of RSCU.


$${RSCU}_{i}=\frac{X_{ij}}{\frac{1}{n_{j}}\sum_{j=1}^{n_{j}}X_{ij}}$$

*X_ij_* is the total codon count of the i^th^ codon of the j^th^ amino acid and *n_j_* is the number of synonymous codons of the j^th^ amino acid. *RSCU_i, max_* is the value of RSCU of the most frequent codon of j^th^ amino acid. The ratio of a codon's observed frequency to the average frequency of synonymous codons is the RSCU value of a codon. If a codon's RSCU score is 1, it means the gene's codon usage pattern is free of synonymous bias. Codons with RSCU values more or less than one, on the other hand, show positive or negative synonymous codon bias in the codon usage pattern, respectively. Uneven use of synonymous codons in genes is a feature of highly expressed genes. A non-zero CAI score indicates a divergence from even use of synonymous codons. The greater the CAI score, the more strongly expressed the genes are thought to be. The relative codon bias (RCB) [25] was devised to quantify the codon bias from the difference between the gene's codon usage pattern and random codon usage [21,23]. When there is no bias, the base composition is random at any codon position. Under the assumption that the base composition is biased at three codon sites, the RCB was calculated by dividing the difference in observed and expected frequency of a codon by the expected frequency [22]. Zero value of RCB indicates no codon bias or random codon usage. When RCB scores greater or less than zero, codons are positively or negatively biased respectively.

The RCB is given by


$${RCB}_{xyz}=\frac{f_{xyz}-{f(x)}_{1}{f(y)}_{2}{f(z)}_{3}}{{f(x)}_{1}{f(y)}_{2}{f(z)}_{3}}$$

where *f_xyz_* represents normalized codon(xyz) frequency. *f(m)_n_* is the normalized base(m) frequency at n^th^ codon site. The influence of natural selection in the codon usage pattern of a gene is indicated by the value of RCB. If the codons have RSCU > 1 and RCB > 0, they are thought to be optimal codons. The rare codons are identified by RSCU < 0.5 and RCB < 0.0.

The modified relative codon bias strength (MRCBS) based on RCB has been developed as an alternative model to predict gene expression level [30-33] and is defined as


$$MRCBS={\left(\prod_{i=1}^{N}\frac{{RCBS}_{i}}{{RCBS}_{i,max}}\right)}^{\frac{1}{N}},$$

where ${RCBS}_{i}=1+{RCB}_{xyz}.$ ${RCBS}_{i,\max\;\;}$ is the maximum value of relative codon bias strength (RCBS) among all codons encoding same amino acid by ith codon(xyz) in the whole genome. The highly expressed gene is characterized on the basis of the strength of the MRCBS provided its value exceeds the threshold value.

The NC-Plot is a conventional NC vs. GC3s curve. It's highly useful to figure out how mutation and selection affect the codon usage pattern of the genes of an organism. The effective number of codons are plotted in respect of standard curve of expected ENC. Expected ENC values of protein-coding sequences have been calculated based on GC3s compositions of the sequences and are given by Chen [34]


$$NC=2+S+\frac{29}{S^{2}+{\left(1-S\right)}^{2}}$$

where S represents GC3s content of the coding sequences.

The effective number of codons (NC) is given by Wright [28],


$$N_{C}=2+\frac{9}{F_{2}}+\frac{1}{F_{3}}+\frac{5}{F_{4}}+\frac{3}{F_{6}},$$

where $F_{k}=(m\sum_{i=1}^{k}\;{\left(\frac{m_{i}}{m}\right)}^{2}-1)/(m-1)$, and m_i_ is the number of occurrences of ith codon for the k-fold degenerate amino acid having total m number of synonymous codons. The value of N_C_ ranges from 20 to 61. The lower values of N_C_ (<35) indicate strong codon usage bias of a gene. When the codon bias of genes is only influenced by the mutational pressure, all the data points will fall on the standard curve (ENC vs. GC3_S_). If the data points are dispersed widely from the standard curve, it indicates that the codon bias is influenced by variables other than mutational pressure.

### Correspondence analysis

The variance in codon usage across genes in different organisms was investigated using correspondence analysis. Excepting methionine, tryptophan, and stop codons, the codon usage of genes are plotted on 59 axes in a multidimensional space [35,36]. In this analysis, the Pearson correlation coefficient was calculated at the level of statistical significance of p < 0.01 to identify the major factors which influence the codon usage variation in different organisms.

## Results

### and Discussion

**Fig. 1.**
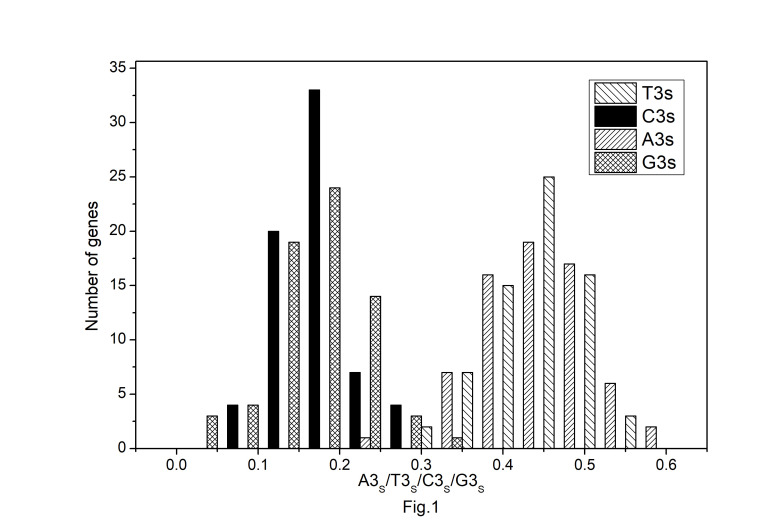
Distribution of nucleotide composition at the synonymous third codon positions in protein-coding genes of *Atropa belladonna* chloroplast genome.

**Fig. 2.**
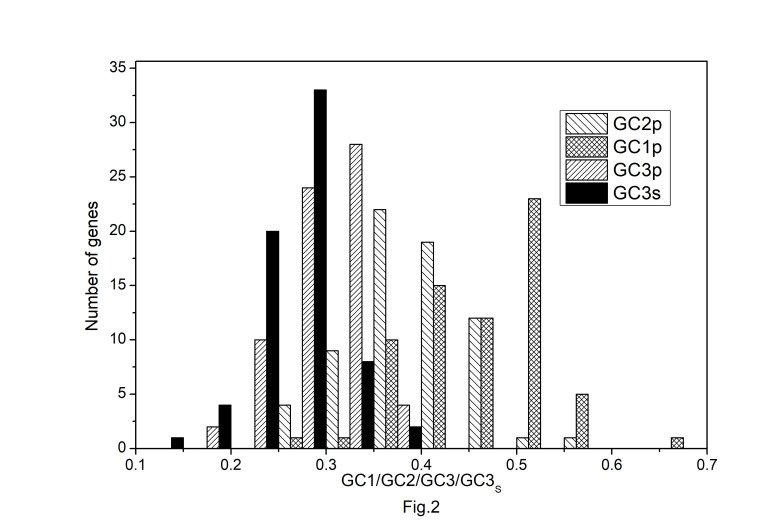
Distribution of GC content at the first, second, and third position of codons in protein-coding genes of *Atropa belladonna* chloroplast genome.

The codon usage pattern in the chloroplast genome of *A. belladonna* and other similar genomes in the nightshade family was investigated in this study. The nucleotide compositions at synonymous and non-synonymous codon sites, and also the dinucleotide composition, have a significant impact on a gene's codon usage bias. In order to better understand the factors that control codon usage in the chloroplast genome, we looked at the overall nucleotide organization and other compositional features at different nucleotide positions in the chloroplast genes of *A. belladonna* and other related genomes in the nightshade family (Table 1). The chloroplast genome of *A. belladonna* is 156,687 bp in length consisting of 85 protein-coding genes, 8 rRNAs, and 37 tRNAs. It has a total GC content of 37.6%, which is similar to that of other nightshade chloroplast genomes [37]. The GC contents at the first, second, and third codon positions in the protein-coding region are 46.37%, 39.66%, and 28.79%, respectively. Genes in the nightshade family's chloroplast genome were discovered to be AT-rich, with AT content at the third codon position being substantially greater than at the other two. At three codon locations, the base composition was discovered to be varied. It demonstrated the presence of selection force in the codon usage, implying that a specific nucleotide may be preferred at three separate codon sites. The nucleotide and dinucleotide organization of the genome has a big impact on whether one form of a codon is preferred over another. The nucleo-bases T and A were discovered to be more common than the nucleo-bases G and C. The same tendency was observed in nucleotide composition at the synonymous third codon position, with T3s and A3s outnumbering G3s and C3s (Fig. 1). The overall base composition of the coding sequences of genes, as well as the composition at the third codon position, revealed that compositional constraints may alter the codon arrangement of genes. The average GC content differed from the GC content of codons in the first, second, and third positions (Fig. 2). The third codon position had less GC content than the first and second codon locations, with the first and third codon positions having the biggest variation in GC content. It's possible that GC content or GC3s have a considerable impact on codon usage patterns and, as a result, on expression profiles. For the investigation of compositional bias, the frequencies of 16 dinucleotides, as well as their anticipated frequencies, were calculated. The identification of favored dinucleotides, as well as the trend in dinucleotide usage may have an impact on codon selection in a gene. Among the dinucleotides, TpT, ApA, and ApT were found to occur more frequently, while CpG, GpC, and CpC were shown to be less common.

### PR2-plot

**Fig. 3.**
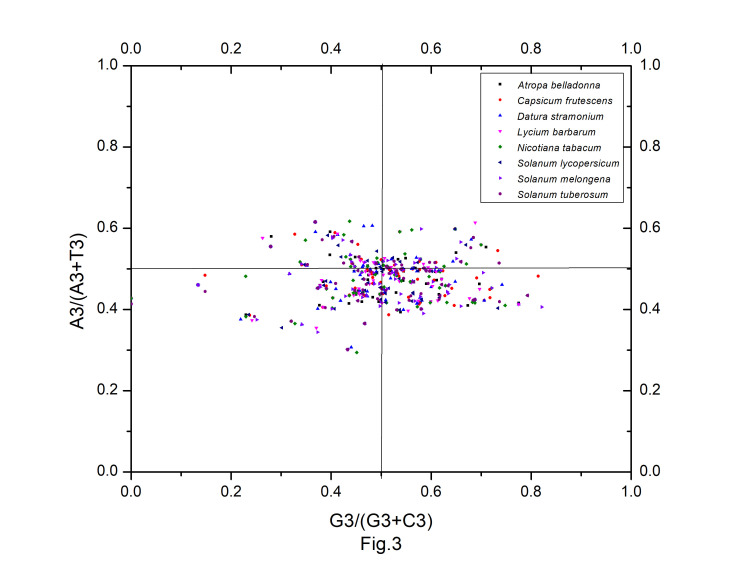
The PR2 plot of protein-coding genes of *Atropa belladonna* and other related chloroplast genomes in the nightshade family.

It was an efficient way to account the influence of the mutation pressure on the codon usage pattern by analyzing the graph of G3/(G3 + C3) vs. A3/(A3 + T3). In this plot, the data points were distributed around the central spot [A = T, C = G(PR2)]. The central spot, described as coordinates of the origin (0.5, 0.5), designates no bias between the influences of mutation pressure and natural selection. The vector from the midpoint shows the extent and direction of PR2 bias acting on individual gene. Mutation pressure and natural selection are the major factors considered to shape the codon usage pattern. In case of mutation pressure, then GC and AT ought to be used proportionally among the degenerate codon groups. Whereas, natural selection for codon choice would not necessarily cause the proportional use of G and C (A and T). The distribution of genes in Fig. 3 indicated that the selection pressure exceeded the mutation in the genes of *Solanaceae* species. In the present study, it was estimated from the plot that the AT-bias measured by the average value of A3/(A3 + T3) in *A. belladonna*, *C. frutescens*, *D. stramonium*, *L. barbarum*, *N. tabacum*, *S. lycopersicum*, *S. melongena*, and *S. tuberosum* were 0.475, 0.474, 0.477, 0.475, 0.481, 0.474, 0.472, and 0.472 respectively, while the respective GC-bias [G3/(G3 + C3) ] were 0.498, 0.503, 0.493, 0.501, 0.496, 0.499,0.499, and 0.503. Thus, we observed T/C bias [A3/(A3 + T3) < 0.5 and G3/(G3 + C3) < 0.5] at the third position of codons of chloroplast genes in *A. belladonna*, *D. stramonium*, *N. tabacum*, *S. lycopersicum*, and *S. melongena*, and T/G bias [A3/(A3 + T3) < 0.5 and G3/(G3 + C3) > 0.5] in *C. frutescens*, *L. barbarum*, and *S. tuberosum*. We also observed that pyrimidines were used more frequently than purines in the chloroplast genes of *A. belladonna*, *D. stramonium*, *N. tabacum*, *S. lycopersicum*, and *S. melongena*. The analysis of PR2-plot (Fig. 3) revealed that not only the mutation pressure but also the natural selection and other factors affected the codon usage pattern of chloroplast genes of *A. belladonna* and other related genomes in the nightshade family. Hence, further analyses are needed to explore the extent of the influencing factors between mutation pressure and natural selection.

### Neutrality plot

**Fig. 4.**
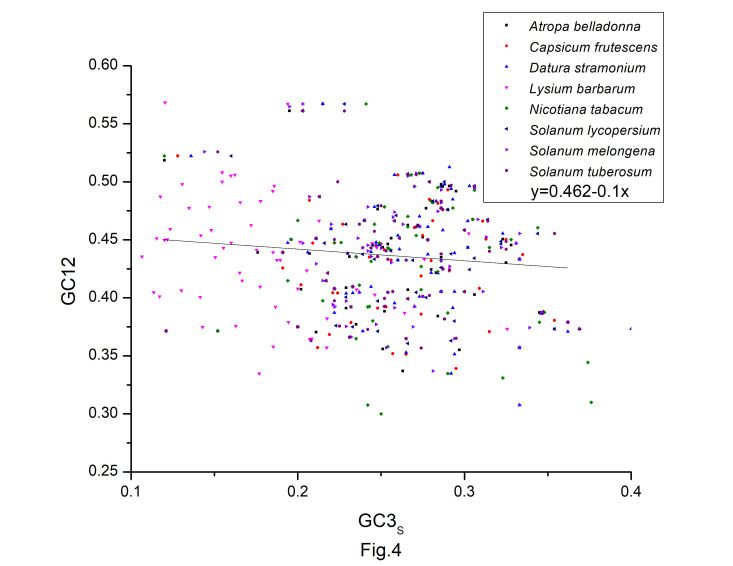
The neutrality plot of protein-coding genes of *Atropa belladonna* and other related chloroplast genomes in the nightshade family.

A neutrality plot (GC12 vs. GC3s) (Fig. 4) was constructed to evaluate the relative impact of mutation pressure and natural selection on codon bias. Natural selection may have influenced codon bias, based on the weak relationships between GC3s and GC12. In the neutrality plot, we noticed that the majority of the genes were placed away from the regression line. According to the slope of the regression lines, relative neutrality (mutation pressure) only accounted for 13.9%, 13.8%, 27.9%, 17.1%, 27.7%, 9.3%, 24.1%, and 5.8%, and the relative constraint on GC3s (natural selection) were 86.1%, 86.2%, 72.1%, 90.7%, 75.9%, and 94.2% in codon usage of *A. belladonna*, *C. frutescens*, *D. stramonium*, *L. barbarum*, *N. tabacum*, *S. lycopersicum*, *S. melongena*, and *S. tuberosum*, respectively. Therefore, during the long evolutionary process, the codons of protein-coding sequences may be more influenced by natural selection and natural selection had a significant impact on the codon usage pattern in *A. belladonna* and other nightshade chloroplast genomes, as evidenced by this study.

### Optimal codons

**Fig. 5.**
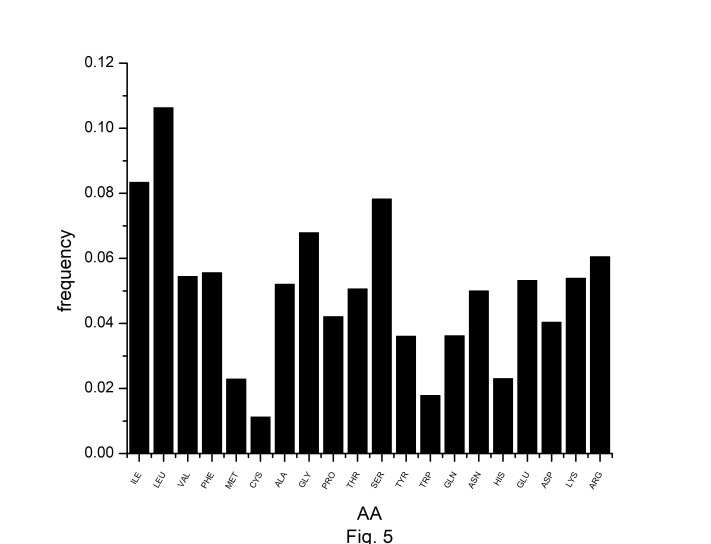
The frequencies of amino acids (AA) in protein-coding genes of *Atropa belladonna* chloroplast genome.

In this study, we have identified optimal codons for analyzing the codon usage and amino acid usage pattern of the *A. belladonna* genome and other chloroplast genomes in the nightshade family. Although most amino acids can be specified by more than one codon, it is hypothesized that in highly expressed genes, only a subset of potential codons is employed. The RSCU has been used to find preferred synonymous codons. The overrepresented codons are identified by using the RCB. The RSCU and RCB of 61 codons are displayed in Table 2. Codons with RSCU greater than 1.0 are favored codons for boosting gene’s translational, whereas codons with RCB greater than zero are overrepresented codons for the organism under study. In *A. belladonna*, the preferred codons are (GCA and GCT) for coding Ala, (AGA, CGA, and CGT) for coding Arg, AAT for Asn, GAT for Asp, TGT for Cys, CAA for Gln, GAA for Glu, (GGA and GGT) for Gly, CAT for His, ATT for Iln, (CTT, TTA, and TTG) for Leu, AAA for Lys, TTT for Phe, (CCA and CCT) for Pro, (TCA, TCT, and AGT) for Ser, (ACA and ACT) for Thr, TAT for Tyr, and (GTA and GTT) for Val. Importantly, these codons reflect a simple compositional bias. Except for TTG of Leu, all the preferred codons have A or T at the third codon position. Whereas, (GCC and GCT) of Ala, (AGA, CGA, and CGT) of Arg, AAT of Asn, GAT of Asp, CAA of Gln, (GAA, and GAG) of Glu, (GGA, GGC, GGG, and GGU) of Gly, (AUC and ATT) of Iln, (TTA and TTG) of Leu, AAA of Lys, ATG of Met, (TTC, and TTT) of Phe, (CCC, and CCT) of Pro, (TCA, TCC, and TCT) of Ser, ACC of Thr, TGG of Trp, and TAT of Tyr are the overrepresented codons. Although RSCU identifies distinct synonymous codons that an organism prefers for translational efficiency in different genes, the set of optimum codons employed in a gene effectively measures the gene's expressivity. The rate of elongation is accelerated by optimal codons, whereas it is slowed by non-optimal codons [38]. In the present study, we observed that GCT of Ala, (AGA, CGA, and CGU) of Arg, AAT of Asn, GAT of Asp, CAA of Gln, GAA of Glu, GGA of Gly, GGT of Gly, ATT of Iln, (TTA, and TTG) of Leu, AAA of Lys, TTT of Phe, CCT of Pro, (TCA, and TCT) of Ser, TAT of Tyr are optimal (RSCU > 1 and RCB > 0). Because of their significant roles in regulating translation elongation, optimum codons have an impact on the stability of mRNA [39]. We estimated the number of each amino acid for all open reading frames across the genome to investigate the amino acid usage pattern in *A. belladonna* genes. The usage of amino acids differed greatly among genes. The chloroplast genome of *A. belladonna* used a lot of leucine, isoleucine, and serine, while histidine, methionine, tryptophan, and cysteine were used very little (Fig. 5). Codons encoding leucine were the most prevalent, accounting for 10.64% of all usage of amino acids. Cysteine-coding codons, on the other hand, were the least common, accounting for only 1.12% of overall usage.

### Correlations among different codon bias indices

**Fig. 6.**
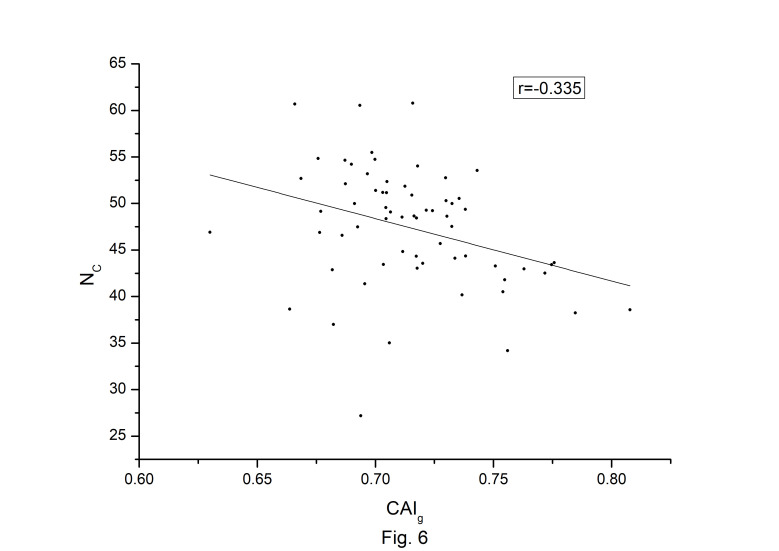
Codon Adaptation Index (CAI) plotted against N_C_ for each protein-coding genes of *Atropa belladonna* chloroplast genome.

**Fig. 7.**
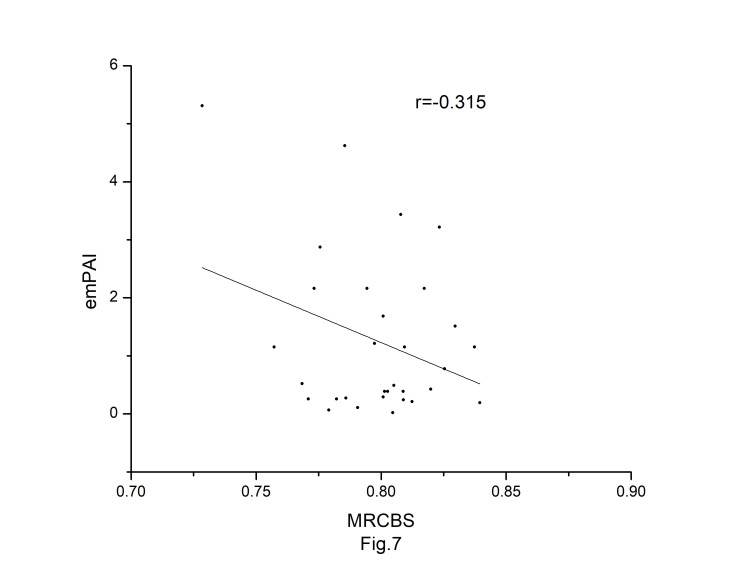
Modified relative codon bias strength (MRCBS) plotted against emPAI [40] for protein-coding genes of *Atropa belladonna* chloroplast genome.

**Fig. 8.**
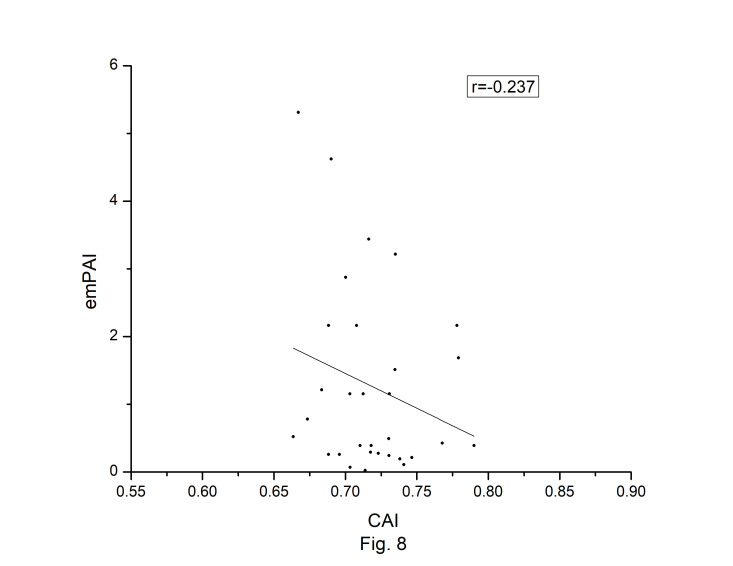
Codon Adaptation Index (CAI) plotted against emPAI [40] for protein-coding genes of *Atropa belladonna* chloroplast genome.

The codon usage bias of the *A. belladonna* and other related genomes in the nightshade family were analyzed in terms of CAI, MRCBS, and Nc. The CAI scores have been calculated in reference to all protein-coding genes. The correlations of the codon usage indices with Nc are very much significant. The correlation of N_C_ with CAI is –0.335. The weak negative correlation between CAI and Nc (Fig. 6) indicates that codon usage bias is low in *A. belladonna*. We observe a strong negative correlation between CAI with GC3_s_ (r = –0.788) (Supplementary Fig. 1), whereas correlation with GC is not much significant (r = –0.231) (Supplementary Fig. 2). So, GC3_s_ not GC content may be the accurate representation of the trend in codon usage bias. The significant correlations of CAI with G3S (r = –0.444) (Supplementary Fig. 3), C3S (r = –0.496) (Supplementary Fig. 4), T3S (r = 0.373) (Supplementary Fig. 5), and A3S (r = 0.253) (Supplementary Fig. 6) indicate the influence of compositional constraint on the codon usage pattern of the genes of *A. belladonna*. The genomic features and different codon usage indices of chloroplast genomes of *A. belladonna* and other seven related plants in the nightshade family have been summarized in Table 3. In order to validate that the gene expressivity measured by codon bias indices like CAI or MRCBS are good indicators for identifying highly expressed genes, we collected proteomic data [40] of chloroplast genome of *Solanaceae* family and compared the results with our predicted values of expression level. Figs.7 and 8 plotted emPAI of chloroplast genes of *N. tabacum* against CAI and MRCBS respectively. Although the data points are scattered, we observed a good correlation between experimental data and predicted results. The agreement of predicted and actual protein expression level varied greatly between all examined combinations of prediction method and data set. The discrepancy is thought to lie in the quality of experimental data. The preliminary analysis on the quality of experimental data shows that these kinds of experiments are inherently noisy and of low reproducibility. The correlation coefficient between emPAI and MRCBS was found to be –0.315 whereas that with CAI was –0.237. The results recommend that a quantitative estimate of expression level may be predicted by codon based indices like MRCBS or CAI.

**Fig. 9.**
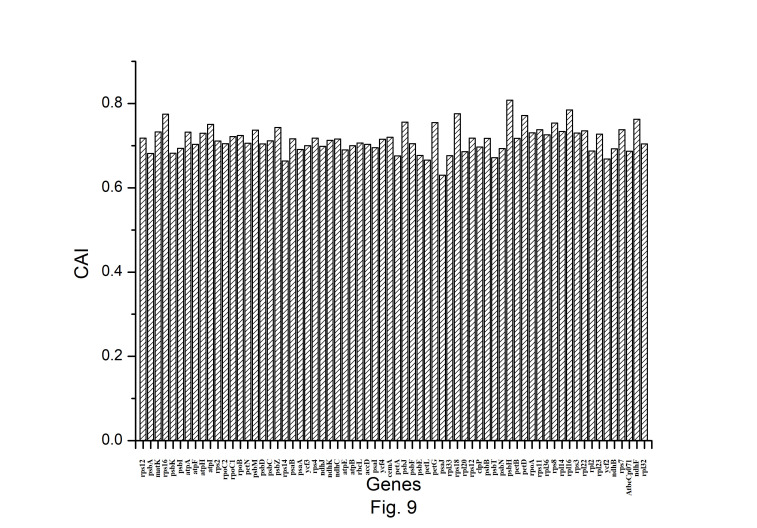
Distribution of Codon Adaptation Index (CAI) of all protein-coding genes of *Atropa belladonna* chloroplast genome.

**Fig. 10.**
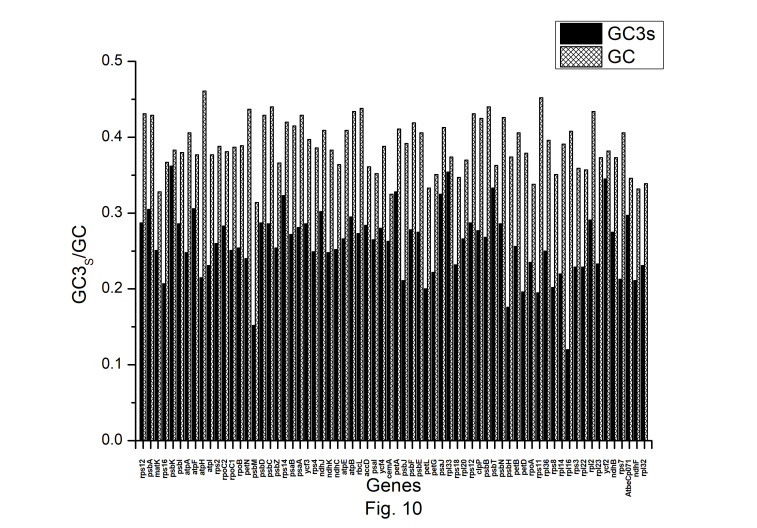
The GC and GC3s content of the protein-coding genes of *Atropa belladonna* chloroplast genome.

The expression profiles of *A. belladonna* chloroplast genes were determined in this work by computing CAI for each gene, and their distributions are presented in Fig. 9. The CAI of the majority of genes (92%) is between 0.66 and 0.78. The z score of CAI values of the gene under study was used to estimate the threshold score for identifying highly expressed genes. The threshold score of CAI has been calculated to be 0.784, and the genes with a z score more than 2.00 are deemed to be PHE genes. Genes with a z score of less than –2.00 are likely to have low levels of expression. Fig. 10 shows the total variation in GC or GC3_s_ content of the genes. It shows that the majority of genes have a GC3_s_ score of 0.120 to 0.362 and a GC content of 0.314 to 0.461.

### NC-plot

**Fig. 11.**
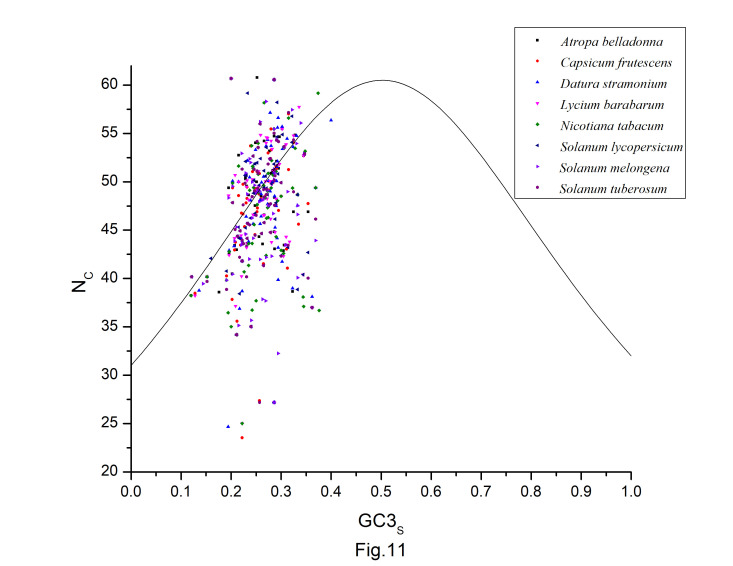
NC-GC3s plot for all protein-coding sequences of *Atropa belladonna* and other related chloroplast genomes in the nightshade family.

NC is a measure of bias in a gene caused by unequal codon use. The inter-genic codon bias is estimated using NC values. The NC of *A. belladonna* genes ranged from 27.18 to 60.77 in our study with a mean value of 47.35 ± 6.23. The smaller the NC value, the more biased a gene is. Codon usage bias is often minimal in *A. belladonna*. N_C_ < 35 is found in just two genes. To clarify the effects of mutation pressure and natural selection, The NC-GC3 plot is created for all protein-coding sequences in the genome to clarify the impacts of mutation pressure and natural selection. Natural selection has a prominent role in defining the codon use variance among those genes, as evidenced by the clustering of points below the anticipated curve (Fig. 11). We also see that some of the data points fall outside of the anticipated curve, implying that additional factors, in addition to natural selection, are likely to have a role in determining the codon usage in *A. belladonna*. Similar trends can be found in the distribution of other related chloroplast genomes in the nightshade family. There are just few points that are near to the curve. Unlike many other chloroplast genomes, the codon usage bias of the chloroplast genomes in the nightshade family is slightly influenced by mutation pressure, but natural selection and other factors may play a large effect. The neutrality plot has also shown that natural selection has a dominant influence on the codon usage pattern.

### Correspondence analysis

**Fig. 12.**
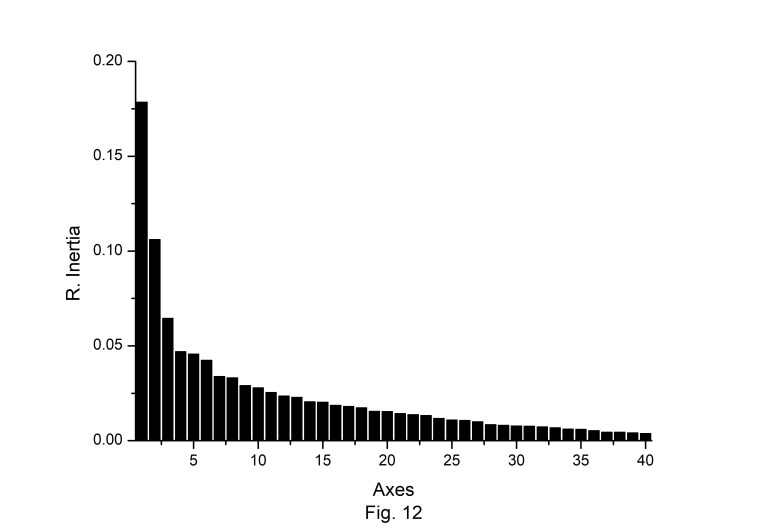
The relative and cumulative inertia of the first 40 factors from correspondence analysis based on the codon usage of *Atropa belladonna* chloroplast genes.

**Fig. 13.**
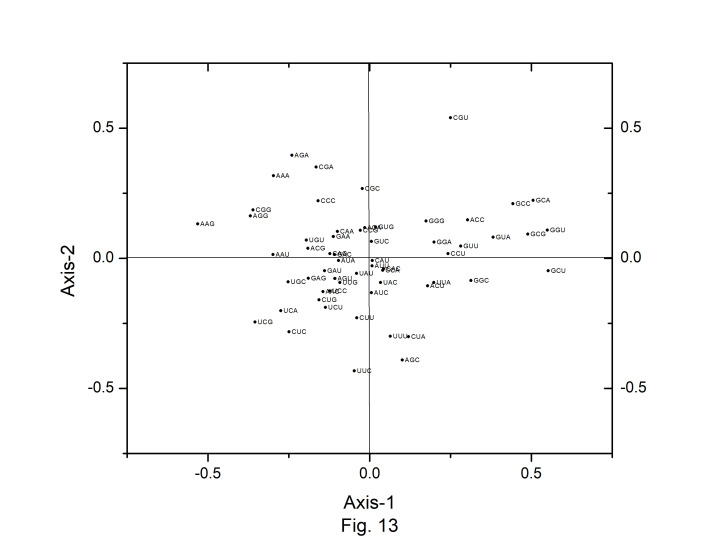
The distribution of codons on axis 1 versus axis 2 in correspondence analysis based on the codon usage of *Atropa belladonna* chloroplast genes.

**Fig. 14.**
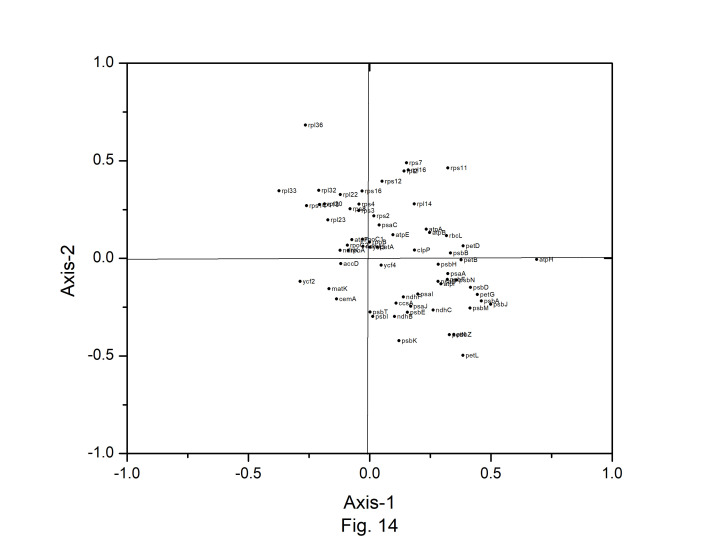
Correspondence analysis of codon usage pattern for chloroplast genes in *Atropa belladonna*.

Correspondence analysis (CA) was performed on the axes generated by the codon usage values of the genes. Only the distributions of the genes along the first two major axes were considered for the present study. The first major axis accounted for 17.89%, 17.19%,17.64%, 17.91%, 15.44%,17.54%, 15.79%, and 17.13% of total variations and the second one for 10.61%, 10.64%, 10.40%, 10.58%, 10.46%, 10.70%, 11.40%, and 10.91% of the total variation in *A. belladonna*, *C. frutescens*, *D. stramonium*, *L. barbarum*, *N. tabacum*, *S. lycopersicum*, *S. melongena*, and *S. tuberosum*, respectively (Fig. 12). Therefore, axis 1 was the major source of variation, responsible for 16%–18% of the total variation. This indicated that the codon usage might be not affected by a single factor. In order to explore the influencing factors that cause variations in codon usage among the genes of *A. belladonna* and other related genomes in the nightshade family, the analyses were performed on commonly used features of protein-coding genes: Gravy, aromaticity, GC3S, GC3 skew, and GC content. In Figs. 13 and 14, codons and genes are plotted against first or second major axes respectively. It has been observed the first principal components are negatively correlated with GC3_s_ (r = −0.349). These findings suggest that highly biased genes, those with G- and C-ending codons, are clustered on the negative side, whereas those with A and T-ending codons predominate on the positive side of the first major axis. The significant positive correlation (r = 0.578) of A3_s_ with second principal components suggested that the highly biased genes with A-ending codons are clustered on the positive sides of the second major axis. The correlation of Gravy and aromaticity score with the first principal components are 0.697 and 0.223 respectively and we observed that the positions of the genes on the axis-2 (2nd principal component) are better correlated with Gravy (r = –0.842) and aromaticity (r = –0.784) compared to the first principal components. Thus, the aromaticity and Gravy score have important roles in determining the codon usages of these genes. We also analyzed the correlation between scores of each of the two axes and levels of gene expression estimated by CAI. The significant positive correlation with CAI (r = 0.369) with first principal components and very weak correlation (r = –0.184) with the second principal components suggested that highly expressed genes are clustered along the positive side of the first major axis. The significant negative correlation (r = –0.362) of A3_s_ and that (r = –0.531) of G3_s_ with first principal components suggested that highly expressed genes with A and G-ending codons are clustered along negative sides of the first major axis.

An important observation in this work is that the hydropathy and aromaticity of the genes affect the codon usage pattern of the chloroplast genes. One of the major axes obtained by CA on the basis of codon usage exhibits a substantial association with the hydropathy and aromaticity of the genes (as measured by the Gravy and Aroma scores) (Table 4). As a result, we observed a significant correlation between nucleotide usage at the third codon sites and the protein hydropathy and aromaticity. We examined the connections between synonymous base usage and hydropathy in the chloroplast genes of *A. belladonna*, and other related genomes in the nightshade family. It is found that hydropathy exhibits the highest negative correlation with A3_s_. In general, hydropathy of the protein-coding genes show positive correlations with T3_s_ and negative with G3_s_ and A3_s_ in *A. belladonna* and other genomes under study. We observed a positive association between hydropathy and C3_s_ (r = 0.224) and a weak negative correlation between hydropathy and G3_s_ (r = –0.157) in *S. tuberosum*. We also looked at the relationship between aromaticity and the use of synonymous bases. Aromaticity is observed to have a positive association with C3S and a negative correlation with A3S. The presence of a significant association between hydropathy and aromaticity of the protein-coding chloroplast genes in *A. belladonna* and other related genomes in the nightshade family, and the base composition at the third codon sites, may influence the physicochemical properties of the protein-coding genes.

### Palindromes and inverted repeats

Palindrome sequences are important in bioinformatics as it helps us to extract patterns in the genomic sequences. The term “palindromes” refers to genetic sequences with reverse-complementary symmetry or the so-called inverted repeat sequences. These significant DNA motifs have been found to have important roles in the control of several cellular processes and are also suspected of being a source of genetic instability. Palindrome sequences are the most common restriction enzyme recognition sites, and they are typically found as important elements in regulatory areas [41]. Inverted repeat sequences in mRNA are also crucial as critical locations of interaction with numerous protein factors involved in the commencement of translation, termination of transcription, and hence gene control [42]. Given the importance of palindrome sequences, a systematic investigation of their occurrence in genome sequences is critical to our understanding of plant evolution. We developed in-house computer software to recognize, locate, and count palindromes in a given sequence for this purpose. The longest palindrome identified in *A. belladonna* AGTTGAA GTACTGAGCCTCCCGATATCGGGAGGC TCAGTACTTCAACT of length 48 (77,046–77,093) is located at the beginning of the gene psbN between the genes psbN and psbT. *C. frutescens*, *D. stramonium*, *L. barbarum*, *N. tabacum*, *S. lycopersicum*, and *S. tuberosum* all had similar palindromes in their chloroplast genomes (Table 5). The other long palindrome of length 40 identified in the *A. belladonna* genome (32,374–32,413) is TTACTTTTTTTATTTAGAAATTTCTAAATAAAAAAAGTAA. Most of the other palindromes of length less than 30 are located in the protein-coding genes.

The longest inverted repeat found in the chloroplast genome of *A. belladonna* TATAAGTGAACTAGATAAAGCGGAATCAAGATTCCGTTTTATCTAGTTCACTTATA of length 56 (79,480–79,535) is located in the intron region of the gene petD and the identical inverted repeats were also found in *C. frutescens*, *D. stramonium*, *N. tabacum*, and *S. tuberosum*. Table 6 lists all inverted repeats with a length of more than 40 found in the *A. belladonna* genome and other nightshade genomes, with the longest inverted GTATAAGTGAACTAGATAAAACG GAATCAAGATTCCGTTTTATCTAGTTCA CTTATAT of length 58 (77,516–77,573) identified in *S. melongena*.

### Phylogenetic study

**Fig. 15.**
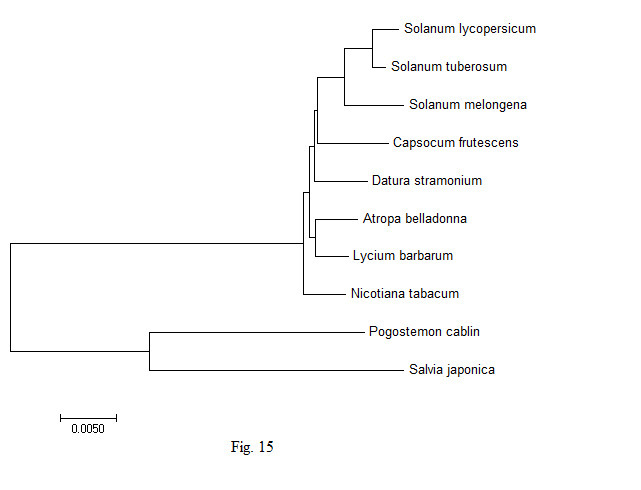
Phylogentic analysis of *Atropa belladonna* and other related genomes in the nightshade family.

In order to understand the genetic variability in the chloroplast genes of the *A. belladonna* and other related genomes in the nightshade family with respect to the nucleotide compositions and to identify the phylogenetic position of *A. belladonna*, a phylogenetic tree analysis was performed for all genomes enrolled in the study. Using the chloroplast genome sequences of *Pogostemon cablin* and *Salvia japonica* as the outgroup species, 70 protein-coding genes of *A. belladonna* and other seven related genomes in the nightshade family were selected for the construction of phylogenetic tree (Fig. 15). CLUSTALW was used to align sequences [43]. Once aligned, phylogenetic trees of the genomes under the present study were constructed by using MEGA 7.0 (https://www.megasoftware.net) with UPGMA [44]. In the present study, *A. belladonna* was found to be isolated from *Solanum* and grouped with *N. tabacum* [45]. Phylogenetic analysis revealed that *A. belladonna* is closer to *L. barbarum* and *N. tabacum* and more distant from *S. melongena*, *S. lycopersicum*, and *S. tuberosum*.

The codon usage pattern in the chloroplast genome of *A. belladonna* and other nightshade plants, as well as the various influencing factors revealed to play a role in determining codon bias, were explored in this work. Natural selection largely influenced the choice of preferred codons and hence codon bias in the chloroplast genes of the nightshade family, according to the analysis of the big data set. The average NC values of protein-coding genes were more than 45, indicating modest CUB in the chloroplast genes of the nightshade family. We observed that the bulk of preferred codons in chloroplast genes of *A. belladonna* and other related genomes in the nightshade family were A3/T3 rich, with only a few genes belonging to PHE genes. We found that there was no significant relationship between GC content and CAI, as evidenced by weak correlation coefficient (r = –0.231) between them. However, a high negative association between GC3 and CAI (r = –0.788) revealed that natural selection had a dominant influence on the codon usage of the chloroplast genome of *A. belladonna* and others in the nightshade family. The codon usage pattern is hypothesized to play a role in regulating gene expression. Natural selection has resulted in a codon bias that improves a gene's translational efficiency by increasing the number of tRNAs present in the genome. CAI was designed to account for codon bias of a gene, allowing it to PHE genes. The low bias of chloroplast genes in *A. belladonna* and others in the nightshade family was suggested by the modest negative correlation coefficient (r = –0.335) between CAI and Nc. In this study, the CA was employed to determine the primary contributing variables in shaping the codon usage pattern of the chloroplast genes in the nightshade family. Phylogenetic tree analysis indicated that the pattern of coding sequences in the chloroplast genome of *A. belladonna* is more closer to *L. barbarum* and *N. tabacum* than it is to *S. melongena*, *S. lycopersicum*, and *S. tuberosum*. The location of long palindrome sequences and inverted repeats in chloroplast genomes were very much significant as they may be identified as essential elements in regulatory regions. The chloroplast genomes of eight nightshade species were analyzed and found to be very similar in terms of gene content and organization. Many of the features in a few genes, on the other hand, were found to be genus- or even species-specific, suggesting that they could be employed as molecular markers to study genetic diversity and evolution. Given the availability of a whole genome sequence, useful information on functional genes may be extracted using computational techniques, laying the groundwork for future research into heterologous protein expression in biotechnological applications. The genetic engineering leading to chloroplast transformation has opened a new era in plant biotechnology.

## Figures and Tables

**Table 1.** The compositional features of chloroplast genomes of Atropa belladonna and other seven related plants in the nightshade family

	*Atropa belladonna*	*Capsicum frutescens*	*Datura stramonium*	*Lycium barbarum*	*Nicotiana tabacum*	*Solanum lycopersicum*	*Solanum melongena*	*Solanum tuberosum*
A	0.2968	0.2953	0.2951	0.2957	0.2992	0.2945	0.2929	0.2943
C	0.179	0.1784	0.1808	0.1794	0.1785	0.1797	0.1792	0.1788
G	0.2081	0.2084	0.2088	0.2093	0.2075	0.2094	0.2076	0.21
T	0.3161	0.3179	0.3154	0.3157	0.3147	0.3164	0.3203	0.3169
A3s	0.422	0.4202	0.419	0.4197	0.4257	0.4177	0.4178	0.4184
C3s	0.1634	0.1626	0.1705	0.1646	0.168	0.1658	0.1654	0.1619
G3s	0.1662	0.1693	0.1693	0.1693	0.1711	0.1693	0.1684	0.1686
T3s	0.4636	0.4646	0.4573	0.4619	0.458	0.4613	0.4642	0.4658
GC	0.3894	0.3889	0.3917	0.391	0.3881	0.3914	0.389	0.3909
GC1	0.4705	0.4662	0.4717	0.4711	0.4653	0.4683	0.4673	0.4698
GC2	0.3995	0.4011	0.3978	0.4008	0.3953	0.4032	0.3979	0.4037
GC3_S_	0.2596	0.2609	0.2675	0.2628	0.2647	0.2641	0.2627	0.2602

**Table 2.** The RCB and RSCU of 61 codons in the chloroplast genome of Atropa belladonna

Amino acid	Codon	RCB	RSCU	Amino acid	Codon	RCB	RSCU
ALA	GCA	–0.151	1.132^a^	LEU	CUG	–0.261	0.41
	GCC	0.204^a^	0.714		CUU	–0.004	1.299^a^
	GCG	–0.470	0.356		UUA	0.336^a^	1.835^a^
	GCU	0.139^a^	1.799^a^		UUG	0.775^a^	1.228^a^
ARG	AGA	0.042^a^	1.804^a^	LYS	AAA	0.382^a^	1.479^a^
	AGG	–0.274	0.633		AAG	–0.035	0.521
	CGA	0.355^a^	1.452^a^	MET	AUG	0.443^a^	1.000^a^
	CGC	–0.229	0.368	PHE	UUC	0.826^a^	0.7111
	CGG	–0.178	0.443		UUU	0.243^a^	1.289^a^
	CGU	0.025^a^	1.300^a)^	PRO	CCA	–0.001	1.163^a^
ASN	AAC	–0.083	0.471		CCC	0.396^a^	0.723
	AAU	0.120^a^	1.529^a^		CCG	–0.052	0.556
ASP	GAC	–0.276	0.403		CCU	0.131^a^	1.559^a^
	GAU	0.077^a^	1.597^a^	SER	AGC	–0.419	0.346
CYS	UGC	–0.484	0.549		AGU	–0.231	1.220^a^
	UGU	–0.487	1.451^a^		UCA	0.016^a^	1.191^a^
GLN	CAA	0.526^a^	1.506^a^		UCC	0.879^a^	0.979
	CAG	–0.006	0.494		UCG	–0.061	0.554
GLU	GAA	0.564^a^	1.487^a^		UCU	0.232^a^	1.710^a^
	GAG	0.071^a^	0.513	THR	ACA	–0.227	1.208^a^
GLY	GGA	0.962^a^	1.590^a^		ACC	0.155^a^	0.804
	GGC	0.078^a^	0.432		ACG	–0.467	0.42
	GGG	0.543^a^	0.7		ACU	–0.153	1.568^a^
	GGU	0.196^a^	1.277^a^	TRP	UGG	1.636^a^	1.000^a^
HIS	CAC	–0.336	0.458	TYR	UAC	–0.302	0.383
	CAU	–0.160	1.542^a^		UAU	0.107^a^	1.617^a^
ILE	AUA	–0.191	0.918	VAL	GUA	–0.261	1.499^a^
	AUC	0.197^a^	0.604		GUC	–0.440	0.505
	AUU	0.100^a^	1.478^a^		GUG	–0.448	0.564
LEU	CUA	–0.289	0.783		GUU	–0.404	1.432^a^
	CUC	–0.093	0.445				

RCB, relative codon bias; RSCU, relative synonymous codons usage.

a RSCU > 1 or RCB > 0.

**Table 3.** The genomic features and codon usage indices of chloroplast genomes of Atropa belladonna and other seven related plants in the nightshade family

Genome	N_C_	Aroma	Gravy	GC (Av)	GC3_AV_	CAI
*Atropa belladonna*	47.35 ± 6.35	0.107 ± 0.045	0.094 ± 0.659	0.390 ± 0.035	0.259 ± 0.045	0.715 ± 0.032
*Capsicum frutescens*	46.99 ± 6.71	0.107 ± 0.047	0.106 ± 0.659	0.389 ± 0.035	0.260 ± 0.046	0.716 ± 0.031
*Datura stramonium*	47.57 ± 7.01	0.107 ± 0.049	0.093 ± 0.644	0.392 ± 0.034	0.267 ± 0.049	0.714 ± 0.032
*Lycium barbarum*	47.12 ± 6.78	0.106 ± 0.048	0.092 ± 0.657	0.391 ± 0.034	0.262 ± 0.046	0.717 ± 0.031
*Nicotiana tabacum*	46.97 ± 6.86	0.107 ± 0.048	0.060 ± 0.668	0.388 ± 0.036	0.265 ± 0.050	0.720 ± 0.033
*Solanum lycopersicum*	47.51 ± 6.17	0.107 ± 0.048	0.102 ± 0.663	0.391 ± 0.035	0.264 ± 0.045	0.717 ± 0.032
*Solanum melongena*	46.90 ± 6.64	0.110 ± 0.046	0.132 ± 0.647	0.389 ± 0.035	0.26 ± 0.049	0.720 ± 0.033
*Solanum tuberosum*	47.44 ± 6.14	0.107 ± 0.046	0.099 ± 0.661	0.391 ± 0.035	0.26 ± 0.045	0.721 ± 0.031

CAI, Codon Adaptation Index.

**Table 4.** Correlations between the protein Gravy/Aroma and synonymous base compositions

	*Atropa belladonna*	*Capsicum frutescens*	*Datura stramonium*	*Lycium barbarum*	*Nicotiana tabacum*	*Solanum lycopersicum*	*Solanum melongena*	*Solanum tuberosum*
Gravy								
A3s	–0.531	–0.484	–0.522	–0.514	–0.516	–0.502	–0.548	–0.516
C3s	0.08	0.105	0.047	0.102	–0.055	0.062	0.049	0.014
G3s	–0.354	–0.368	–0.368	–0.357	–0.362	–0.391	–0.340	–0.384
T3s	0.272	0.227	0.315	0.262	0.334	0.247	0.318	0.301
Aroma								
A3s	–0.440	–0.375	–0.388	–0.402	–0.307	–0.373	–0.370	–0.387
C3s	0.376	0.377	0.488	0.406	0.376	0.361	0.465	0.305
G3s	–0.062	–0.056	–0.035	–0.060	0.047	–0.071	–0.087	–0.061
T3s	0.227	0.168	0.102	0.183	0.129	0.145	0.089	0.202

**Table 5.** Identification of palindromes of length greater than 30 in the chloroplast genomes of *Atropa belladonna* and other related plants in the nightshade family

Genome	Palindrome	Length	Location	Region
*Atropa belladonna*	AGTTGAAGTACTGAGCCTCCCGATATCGGGAGGCTCAGTACTTCAACT	48	77,046–77,093	psbT-psbN
*Atropa belladonna*	TTACTTTTTTTATTTAGAAATTTCTAAATAAAAAAAGTAA	40	32,374–32,413	tRNA(Glu)-tRNA(Thr)
*Lycium barbarum*	AGTTGAAGTACTGAGCCTCCCGATATCGGGAGGCTCAGTACTTCAACT	48	76,719–76,766	psbT-psbN
*Capsicum frutescens*	AGTTGAAGTACTGAGCCTCCCGATATCGGGAGGCTCAGTACTTCAACT	48	77,542–77,589	psbT-psbN
*Solanum lycopersicum*	AGTTGAAGTACTGAGCCTCCCGATATCGGGAGGCTCAGTACTTCAACT	48	76,202–76,249	psbT-psbnN
*Solanum melongena*	GTTGAAGTACTGAGCCTCCCGATATCGGGAGGCTCAGTACTTCAAC	46	75,091–75,136	psbT-psbN
*Datura stramonium*	AGTTGAAGTACTGAGCCTCCCGATATCGGGAGGCTCAGTACTTCAACT	48	76,485–76,532	psbT-psbN
*Solanum tuberosum*	AGTTGAAGTACTGAGCCTCCCGATATCGGGAGGCTCAGTACTTCAACT	48	76,042–76,089	psbN-psbN

**Table 6.** Identification of inverted repeats of length greater than 30 in the chloroplast genomes of *Atropa belladonna* and other related plants in the nightshade family

Genome	Inverted repeats	Length	Location	Region
*Atropa belladonna*	TATAAGTGAACTAGATAAAGCGGAATCAAGATTCCGTTTTATCTAGTTCACTTATA	56	79,480–79,535	petD (intron)
*Atropa belladonna*	GAGAGCTCGGATCGAATCGGTATTGATATACCGATTCGATCCGAGCTCTT	50	146,901–146,950	tRNA (Leu)-ycf2
*Atropa belladonna*	AGAGCTCGGATCGAATCGGTATATCAATACCGATTCGATCCGAGCTCT	48	96,608–96,655	ycf2-tRNA (Leu)
*Lycium barbarum*	GAGAGCTCGGATCGAATCGGTATTGATATACCGATTCGATCCGAGCTCTT	50	145,879–145,928	tRNA (Leu)-ycf15
*Lycium barbarum*	AGAGCTCGGATCGAATCGGTATATCAATACCGATTCGATCCGAGCTCT	48	96,284–96,331	ycf15-tRNA (Leu)
*Capsicum frutescens*	TATAAGTGAACTAGATAAAACGGAATCAAGATTCCGTTTTATCTAGTTCACTTATA	56	79,975–80,030	petD (intron)
*Solanum lycopersicum*	TAAGTGAACTAGATAAAAGGGAATCTTGATTCCCTTTTATCTAGTTCACTTA	52	78,626–78,677	petD (intron)
*Solanum melongena*	GTATAAGTGAACTAGATAAAACGGAATCAAGATTCCGTTTTATCTAGTTCACTTATAT	58	77,516–77,573	petD (intron)
*Datura stramonium*	TATAAGTGAACTAGATAAAACGGAATCTTGATTCCGTTTTATCTAGTTCACTTATA	56	78,906–78,961	petD (intron)
*Datura stramonium*	GAGAGCTCGGATCGAATCGGTATTGATATACCGATTCGATCCGAGCTCTT	50	146,149–146,198	tRNA (Leu)-ycf15
*Nicotiana tabacum*	TATAAGTGAACTAGATAAAACGGAATCAAGATTCCGTTTTATCTAGTTCACTTATA	56	79,243–79,298	petD (intron)
*Nicotiana tabacum*	GAGAGCTCGGATCGAATCGGTATTGATATACCGATTCGATCCGAGCTCTT	50	146,240–146,289	tRNA (Leu)-ycf2
*Nicotiana tabacum*	AGTTGAAGTACTGAGCCTCCCGATACCGGGAGGCTCAGTACTTCAACT	48	76,815–76,862	psbT-psbN
*Nicotiana tabacum*	AGAGCTCGGATCGAATCGGTATATCAATACCGATTCGATCCGAGCTCT	48	96,342–96,389	ycf2-tRNA (Leu)
*Solanum tuberosum*	TATAAGTGAACTAGATAAAAGGGAATCAAGATTCCCTTTTATCTAGTTCACTTATA	56	78,473–78,528	petD (intron)
*Solanum tuberosum*	GAGAGCTCGGATCGAATCGGTATTGATATACCGATTCGATCCGAGCTCTT	50	145,621–145,670	tRNA (Leu)-ycf2
*Solanum tuberosum*	AGAGCTCGGATCGAATCGGTATATCAATACCGATTCGATCCGAGCTCT	48	95,365–95,412	ycf2-tRNA (Leu)

## Supplementary Materials

Supplementary Fig. 1. CAI plotted against GC3S for each protein-coding genes of *Atropa belladonna* chloroplast genome.

Supplementary Fig. 2. CAI plotted against GC for each protein-coding genes of *Atropa belladonna* chloroplast genome.

Supplementary Fig. 3. CAI plotted against G3s for each protein-coding genes of *Atropa belladonna* chloroplast genome.

Supplementary Fig. 4. CAI plotted against C3s for each protein-coding genes of *Atropa belladonna* chloroplast genome.

Supplementary Fig. 5. CAI plotted against T3s for each protein-coding genes of *Atropa belladonna* chloroplast genome.

Supplementary Fig. 6. CAI plotted against A3s for each protein-coding genes of *Atropa belladonna* chloroplast genome.
