# Supplementary figures and images for: The pattern of coding sequences in the chloroplast genome of Atropa belladonna and a comparative analysis with other related genomes in the nightshade family
Source: Genomics Inform. 2022 Dec 26;20(4):e43. doi: 10.5808/gi.22045 (PMC9847383; doi:10.5808/gi.22045)

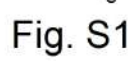

Supplement: Supplementary Fig. 1. — CAI plotted against GC3S for each protein-coding genes of Atropa belladonna chloroplast genome. [file gi-22045suppl1.pdf]

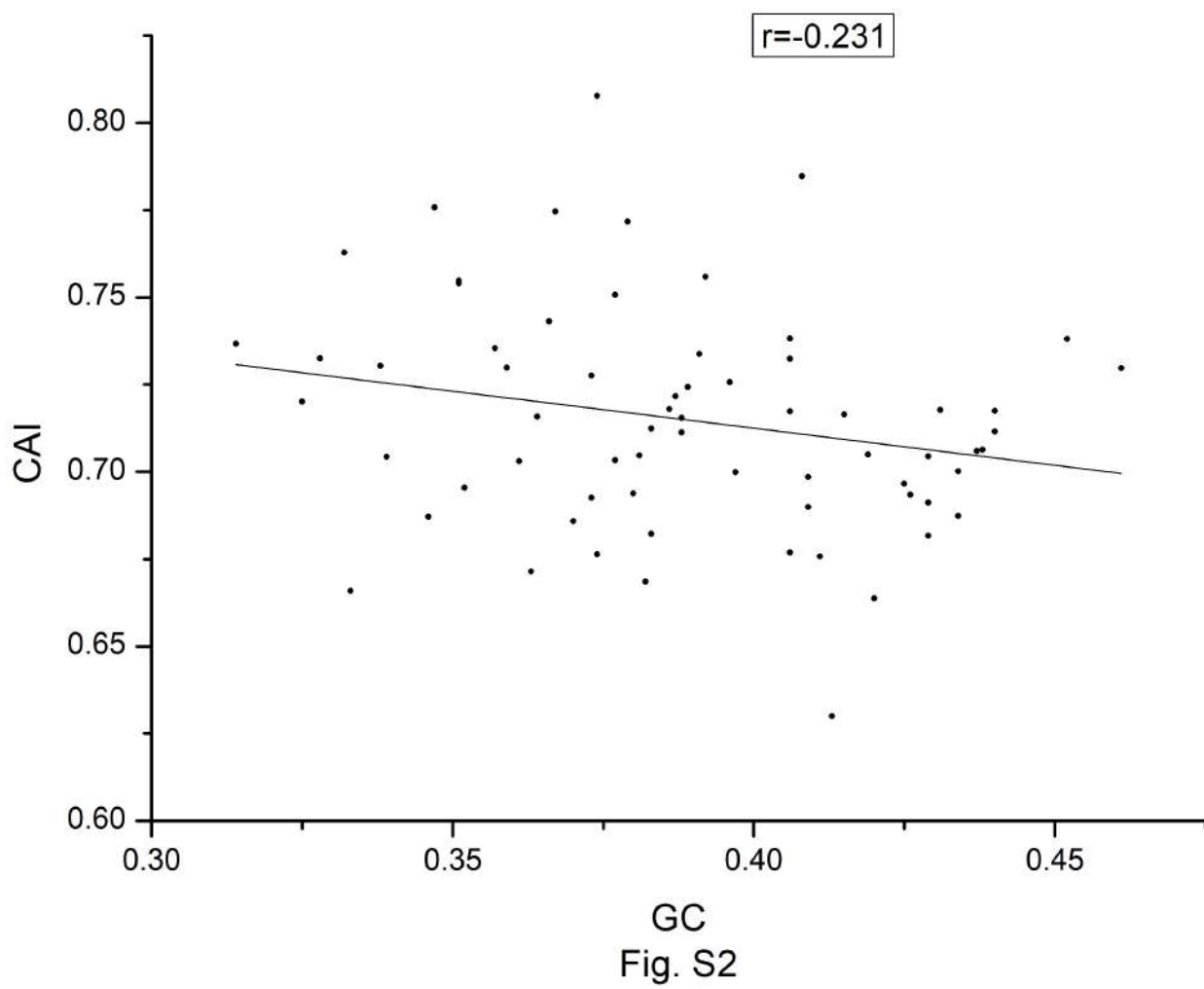

Supplement: Supplementary Fig. 2. — CAI plotted against GC for each protein-coding genes of Atropa belladonna chloroplast genome. [file gi-22045suppl2.pdf]

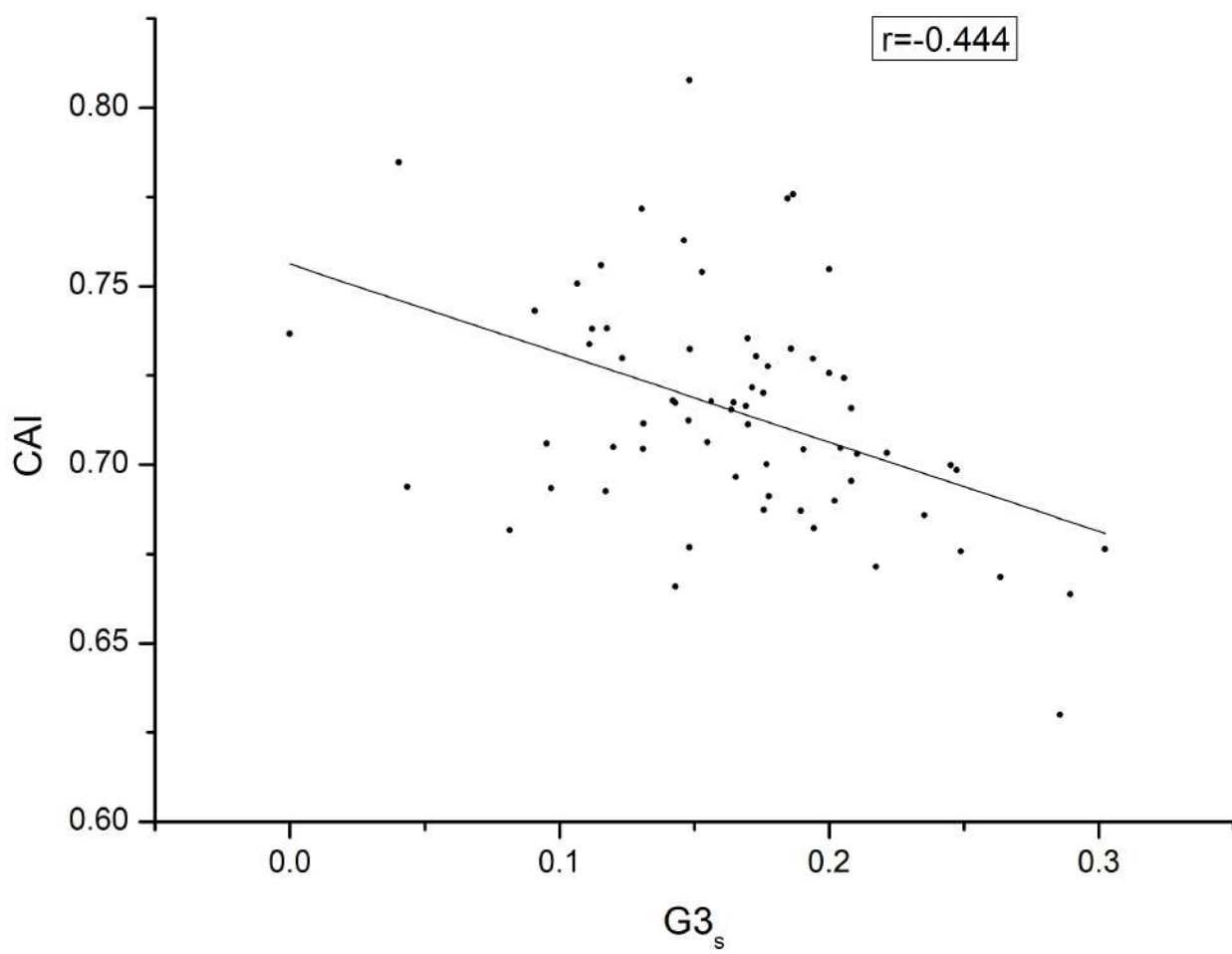

Fig. S3

Supplement: Supplementary Fig. 3. — CAI plotted against G3s for each protein-coding genes of Atropa belladonna chloroplast genome. [file gi-22045suppl3.pdf]

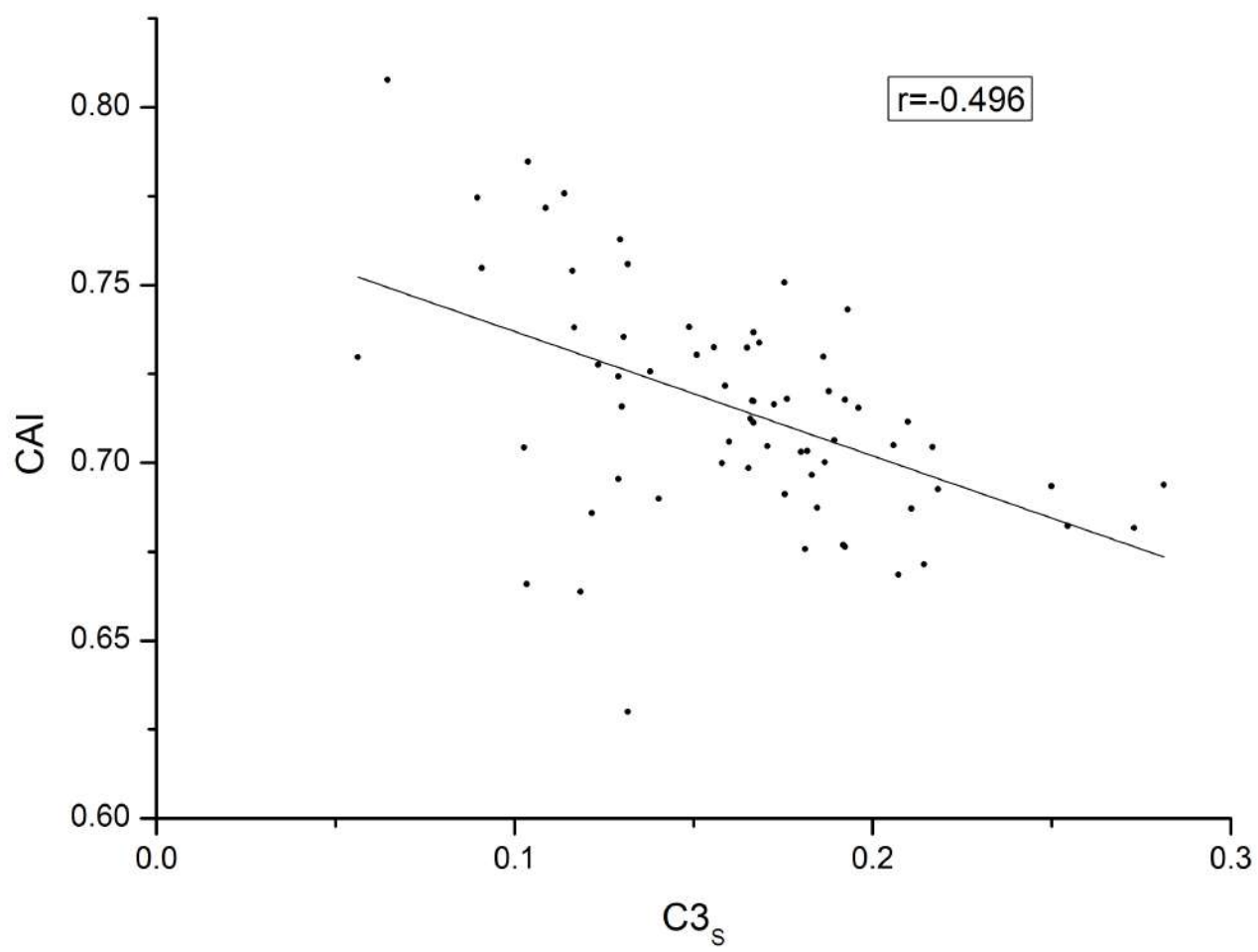

Fig. S4

Supplement: Supplementary Fig. 4. — CAI plotted against C3s for each protein-coding genes of Atropa belladonna chloroplast genome. [file gi-22045suppl4.pdf]

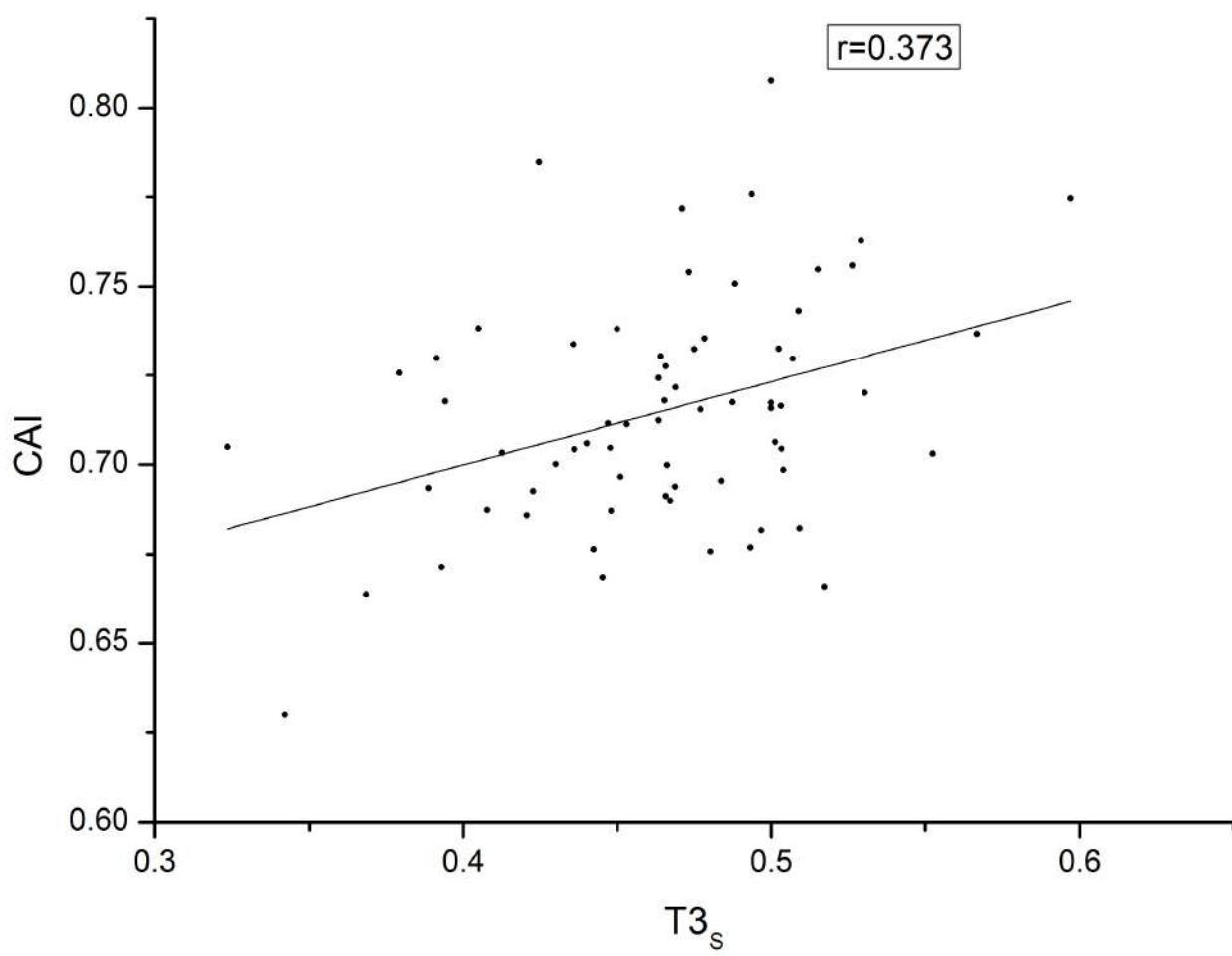

Fig. S5

Supplement: Supplementary Fig. 5. — CAI plotted against T3s for each protein-coding genes of Atropa belladonna chloroplast genome. [file gi-22045suppl5.pdf]

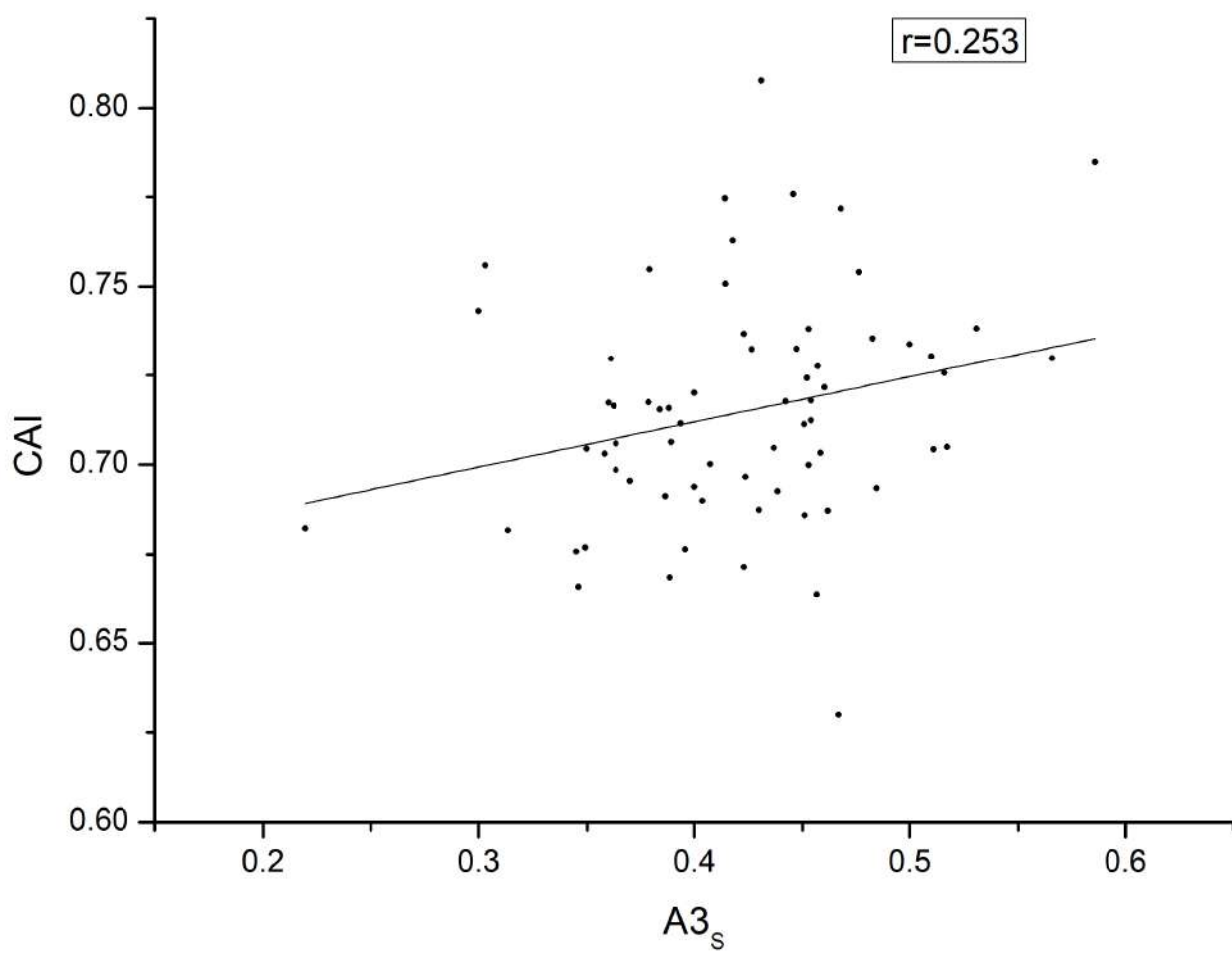

Fig. S6

Supplement: Supplementary Fig. 6. — CAI plotted against A3s for each protein-coding genes of Atropa belladonna chloroplast genome. [file gi-22045suppl6.pdf]
